# Cases and Observations, Illustrating the Origin of Tubercles

**Published:** 1829-01-01

**Authors:** 


### Cases and Observations, illustrating the Origin of Tubercles. By *W. P. Alison*, M.D. Professor of the Institutes, &c.

[Medico-Chirurgical Transactions, Edinburgh.]

Perhaps there is no disease so interesting to a native of the British Empire as phthisis. Whether it be studied as a curiosity, or as a mystery; as a subject involving the welfare of nations, or the safety of individuals, every mind must be alive to its importance. Its blight is not confined to the hovels of poverty, nor does it hold sacred the abodes of temperance. Manhood succumbs to its influence, and youth bends under its withering grasp. It loves to revel in the arms of beauty, and, leaving the old and the infirm to sink, in the quiet course of nature, under the pressure of their years, it luxuriates, with indiscriminating wantonness, amid the fairest and the finest of our species. To dissect a monster of such savage propensities—to study every shade of his minutest habitudes—to unravel the mysteries of his mechanism, and to unfold, as far as possible, the secrets of his constitution and the principles of his system, must be a desideratum in every philanthrophic breast, and every attempt at its execution should be hailed with encouragement and approbation.

From 1796 to 1799, the bills of mortality for London gave 17,559 as its proportion of deaths from consumption, out of a sum total of 52,237, excluding casualties and still-born children. In a table, furnished by Bayle, the following are the proportionals, during three years. From the 22d of March, 1803, to the 21st of March, 1804, out of 300 deaths, 99 were consumptive cases;—from the 23d of September, 1804, to the 22d of September, 1805, out of 228 deaths, 73 were occasioned by phthisis;—from the 1st of January to the 31st of December, 1806, out of 168 deaths, there were 72 consumptions. Out of 696 deaths, therefore, 244 were occasioned by this disease. From these two statements, more than one-third appear to die of consumption; and although the same proportion will not hold in all seasons and in all years, we may, perhaps, without much exaggeration, fix it at one-fourth in our large towns, and one-fifth in country practice. The mortality from consumption, in England alone, would, consequently, amount to 55,000 a year;* and, when we extend our view to other portions of the globe, especially to the other departments of the British Empire and to the Continent, and take into our estimate the mortality in each, the sum total would be perfectly appalling. Such a calculation proves the awful share which consumption holds in the lottery of life, and that, were we to devote one-fourth of our time and talents in elucidating its pathology, and in improving its treatment, it would merely receive its proper dividend of our attention.

While the causes of phthisis are various, tubercles in the lungs are both the most common and the most important. Out of 900 patients labouring under various kinds of this disease, 624 were affected with the tubercular form. Dr. Willan considered that one-fourth of all the cases which occurred in London arose from this cause; and some have gone so far as to maintain, that every pure instance of this disease originates in these morbid depositions. But how these

* It is at least 60,000 at the present moment.—*Ed.*

tubercular bodies are formed—whether they are secreted like common organic matter, in the natural course of the circulation, or are the proceeds of diseased action—whether their formation requires some favourable condition or predisposition in the system to their development, or may result from undue excitement in any texture, under any circumstances—whether their existence be limited to a specific kind of structure, and depend on a specific kind of constitution, or may be found in every structure, may be elicited in every constitution, and, in short, may be subject to all the laws and liberties of organic life—are questions of interesting moment, both in a physiological and practical view.

Hippocrates ascribed their formation to the putrefaction of phlegm, or bile; Sylvius believed they were conglobate glands, and that they existed in profusion within the pulmonary texture; Van Swieten considered all consumptions as arising from a conversion of the blood and chyle into pus; Willis and Hoffman imagined that they resulted from an acrimonious state of the fluids; Haller describes tubercles as either diseased bronchial glands, or vesicles full of viscid humour; Reid considered that they originated in obstruction of the exhalent vessels of the lungs; Rush says they are not glands, but simply collections of inorganic matter, resembling mucus; Baron maintains their hydatid origin; and many other speculations have been indulged in, which it would be only tedious to detail. Our present author takes a different, although not a new view of their formation, and while, perhaps, the nature of the subject he investigates precludes the possibility of direct proof, his arguments and illustrations constitute an evidence in its favour, which must be regarded as much stronger than presumption.

The communication opens with observing that—

"The inquiries on the subject of scrofulous diseases, of which I formerly laid an account before the Society, were directed chiefly to the elucidation of the two following questions: 1*st*, Whether the scrofulous diathesis, or disposition to scrofulous disease, unfortunately so prevalent in our climate, is to be regarded as the result of the climate alone; or whether other and more remediable circumstances in the mode of life of those who become so affected, contribute generally and powerfully to its production; and 2*dly*, Whether the deposition of tubercles, so often the original cause of danger and death in scrofulous diseases, is to be regarded, in any case, or in any considerable number of cases, as an effect of inflammatory action." 274.

As to the first question, he is inclined to believe that we are disposed to attribute too much influence to climate, and too little to the habitudes of social life; and that there is a much greater difference, as to tendency to scrofulous diseases, "between the inhabitants of large towns and of the country, than between the inhabitants of the warm and cold climates, at least of Europe; and, therefore, that the general prevalence of the scrofulous diathesis is to be ascribed, rather to the modes of life which an advanced and artificial state of society implies, than to the circumstance of climate."

It is certainly undeniable, that, in Russia, consumption is rare; in Iceland, according to Hooker, it is not frequent; in Greenland, the mortality it occasions is small; and that in Canada, it is seldom met with. These facts countenance, so far, the Professor's position; but it is equally well known, that, in Persia, pulmonary phthisis is a rare occurence; that it is scarcely known in the West Indies; that it is uncommon in Bengal and Egypt; and that, in Sicily, Malta, and the other Mediterranean Islands, it is not often seen. Warm or temperate climates seem, therefore, unfavourable to its propagation, and we have every reason for anticipating, in theory, what history establishes as fact. Cold is the favourite stimulus of the bronchial lining, and modifies its secretions more than any other form of excitement; and a bland and equable climate can do more in alleviating pulmonary disease, when it has arrived at a certain stage, than all the resources of art. While we admit, therefore, the influence of the artificial habitudes of life in deranging the general health, and in diseasing the pulmonary system, we cannot give up the part which climate acts in producing phthisis, nor even allow to its operation a subordinate effect.

"In regard to the second point, I should wish it be remembered, that my object was, not to explain the whole history, and all the possible causes of tubercles, but only to investigate the question, which seemed of the greatest practical importance; whether or not inflammatory action has, in any circumstances, the power to produce them? And on this subject I cannot help remarking with satisfaction, that although, at the time when I wrote my former paper, the most celebrated authors and teachers in the school of Paris, who had studied this subject minutely, Bayle, Laennec, Rostan, Louis, Velpeau, &c. were disposed to allow little or no influence to inflammation in the production of tubercles; yet those of the French pathologists who have attended most minutely to the subject since that time, particularly Professors Andral and Cruveilhier, have adopted an opinion almost identical with that which I endeavoured to explain." 276.

In the paper to which he alludes in this passage, and of which we gave a full digest shortly after its appearance, he advanced several observations, tending to establish the principle he now undertakes to defend, and supported them by nine cases, to which he subjoins, as a summary of their object and result, the following remarks:—

"I have given these cases, because particular circumstances led me to take short notes of them at the time when they occurred; but I have seen several others, the leading particulars of which were just the same; and I think I can say with confidence, that among the children of the lower orders in this town the case is not a rare one. A healthy child shall be seized suddenly with well-marked pneumonic symptoms, either from cold, or from the contagion of hooping-cough or measles. The symptoms shall be such, as to justify a confident expectation (founded on dissections of children dying in the height of such affections, of which I could produce many examples) that if speedily fatal, they will leave behind them the indications of active inflammation. The urgent danger shall be got over, but the cough and shortness of breath shall continue with progressive emaciation and debility, till death takes place, at the end of some weeks or a few months; and on dissection, there shall appear tubercles in great numbers, and sometimes of a pretty large size, with no condensation of the pulmonary substance, or only a slight condensation of it, just about the tubercles." 431.

The great difficulty which presents itself in the investigation of such a subject, is to collect unequivocal evidence, that the tubercles found upon dissection, and to which death was ascribed, did not exist previous to, and merely accompanied, the attack of inflammation which was supposed to have excited them. It is a well-known and admitted fact, that tubercles may exist in the pulmonary texture for many years, without deranging, to any visible amount, the general health, or betraying their presence by any pulmonary symptom, at least, so palpably pronounced as to excite attention. How often do we discover them in the lungs of those who have died of some very different disease, and how frequently do those whose prejudices, spurning every innovation as a gross reflection on the wisdom of antiquity, denounce, as quackery, the application of the stethoscope, search for them in vain, after having accused them as the sum and substance of all the mischief!

To parry the force of this difficulty the Dr. maintains not that the previous good health of his patient is an unequivocal evidence of the absence of tubercles, but insists upon a connexion between the supervention of pulmonary inflammation to an evident exciting cause, and the existence of tubercles in the lungs after death, as the only morbid products they presented. A child, being in a perfectly good state of health, having neither cough, nor dyspnœa, nor deranged pulse, nor, in short, the slightest sense of pectoral uneasiness, and who, moreover, has never had any preceding attack of inflammation within the chest, receives an injury upon the breast, which is immediately followed by hurried breathing, quick pulse, pain on inspiration, and other symptoms of thoracic disorder, which, gradually increasing, ultimately destroy life. This body is inspected, and within the chest nothing unnatural can be seen, save a crop of tubercles, imbedded within the parenchyma of the lungs, without either inflammation, adhesion, condensation, or effusion. Now, were the tubercles the cause of death, and were they elicited by the inflammation excited by the injury; or was their existence a mere coincidence with the attack of inflammation, having neither the relation of cause or effect to it? The post-mortem results, we presume, answer the first question; for, since no traces of pulmonary disease were visible, except the tubercles, we have no authority to overlook a palpable misstructure, and ascribe death to an undefined something of whose existence we have no proof, indeed, to an absolute nothing. On this supposition we have the lungs stuffed with tubercles without producing any symptom, and we have death *occasioned without a cause!*

But the second question is of a more difficult nature, in as far as it is surrounded by many sources of error, and scarcely admits of direct evidence. It is generally admitted that scrofulous diseases are hereditary, and the facts, by which such an opinion is supported, are so numerous and unquestionable, that no doubt can be entertained as to its accuracy. But we fear that the advocates of this doctrine go rather far when they maintain, that not only a propensity to scrofulous disease is communicable from parent to child, but that the disease itself is substantially implanted, and that the fœtus in utero is often as much diseased as the mother who bears it. They maintain, that tubercles have been found in the lungs of still-born children, nay, some (as Rollo) think it sufficient for the maintenance of their opinion to adduce cases in which tubercles were found some years after birth, forgetting that children are as subject to inflammation (if not more so) as adults. Now, although we pretend not to dispute the accuracy of West, or Orfila, nor to deny the possibility of such an occurrence; we have many reasons for believing that it is very rare. After 139 dissections of still-born, and very young children, Dr. Denis concludes, that they who have described tubercles in the lungs during the fœtal state, had been mistaken,—that he has never seen them "*avant les premiers mois que suivent la naissance*,"—and that they only become visible in the lungs of children who have been *five or six months* old. Velpeau's testimony adds weight to this statement, and Breschet, who being surgeon to the *Enfans Trouvés* of Paris, has large opportunities for observation, has frequently looked for them in vain. But, admitting that they do occasionally occur in the fœtus, we have not indisputable evidence that they have been implanted in the natural course of deposition during the fœtal growth. Children in the womb are susceptible of disease, and disease of a very active character. They have been born with symptoms of bronchitis, and pneumonia, and we know an accoucheur of Metropolitan celebrity, who attributes many of those miscarriages and abortions, which we account for on maternal causes, to idiopathic fœtal diseases. Why may not these tubercles, therefore, when they do appear in the lungs of still-born children, be the effect of inflammatory action, if inflammation can in any case account for their formation; and why should a concession of the principle, that a scrofulous diathesis is hereditary, entitle us to maintain the communicability of scrofulous disease? In support of this explanation we may observe, that Magendie has always discovered traces of inflammation accompanying those tubercles which he has detected in the bodies of young children, and that even in their earliest stage, and minutest form, they were surrounded with obvious vascularity. Whether we admit, therefore, or deny the possibility of tubercles existing in the lungs of the unborn child, we have no reason for adopting the doctrine, that these formations are as hereditary as the principle which forms them; for, if we admit the possibility (and we do not wish to deny it) we can explain the phenomenon without it, and if we discredit it, no explanation is required.

Dr. Alison has remarked that in cases of young children, which have come under his observation, when tubercles have been found in unusual numbers, and have been, to all appearance, the cause of death, "the first and chief symptoms of the disease have been decidedly those of inflammation, from which, the patients never recovered, and of the consequence of which, judging from the symptoms, it was obvious that they died." 279-80. The succeeding case we adduce as an illustration.

A boy, æt. 26 months, who had never had any of the contagious diseases to which children are subject, nor, indeed, any sickness of *a day's duration*, until the 12th of May; when, from exposure to cold, he was seized with heat, restlessness, cough, and short breathing. (13th.) Pulse quick and full, face flushed, skin hot, and breathing much oppressed. The application of eight leeches somewhat relieved him, and by repeating them, taking some laxative medicine, and small doses of tartar emetic, by the end of the first week, the pectoral symptoms were much abated. Some cough, however, remained, his pulse was quick, and his respiration shorter than natural. About this period constipation, a tendency to coma, and slight spasms, excited some suspicion of hydrocephalus; but these symptoms gradually disappeared, and were succeeded by fits of chilliness, followed by heat and thirst. His tongue was rather dry, his appetite bad, and his body emaciated; and, as his respiration became more difficult about the fourth week of his illness; leeches were applied, but without relief, and he died on the 27th day of his disease.

*Dissection.* Left lung condensed, having on its surface flakes of yellow lymph, and when cut into, it presented portions of red hepatization; but the greater part consisted of yellow matter partly *infiltré* through the substance of the lung, partly disposed in roundish tubercular masses, and partly in irregular flakes, resembling, both in form and substance, those upon the surface of the pleura. Some of these tubercles, contained pus, and some were nearly empty. The right lung was large, slightly condensed, but contained few tubercles. The other viscera were healthy. p. 280-81-82.

Now, in this case the boy had enjoyed a perfect state of health, previous to the 12th of May, unmarred by indisposition, and uninterrupted by pain;—no symptoms of pectoral disease could be observed, and no scrofulous action was apparent in the constitution. He was exposed, however, to an exciting cause, and to that cause of all others most calculated to awaken pulmonary inflammation—cold; and, no sooner was the exposure sustained, than signs of thoracic mischief appeared. The date and origin of the attack are, therefore, quite evident, and well marked, and the effect succeeds the cause as naturally and as speedily as could be wished, to prevent confusion. It may, however, be said, that although all this is quite clear, tubercles may have existed prior to the operation of the exciting cause, and that the boy's exposure to cold called these latent bodies into action. But dissection does not countenance such a view. We have almost every relic, which inflammatory action can produce, in the left lung—effusion of lymph, hepatization, tubercular infiltration, distinct tubercles, and vomicæ. No line of separation could be drawn between the hepatized and tuberculated lung—they passed *sine forma* into each other, and the yellow substance, which was thrown out upon the pleura, and which no one will suppose could have existed there in a *latent* state during health, was the same both in appearance and texture, as far as sense could judge, with that tubercular infiltration which we have described as joining the distinct and isolated tubercles, with the inflamed and consolidated lung. This informal and unceremonious transit of one structure into another, we regard as strong evidence of the orthodoxy of this doctrine, and we do not despair of seeing the professor's anticipation realized, as expressed in the following paragraph.

"The gradual and imperceptible transition of the matter constituting tubercles, into that which is clearly the effect of inflammation, is, of all the phenomena observable on dissection, that which appears to furnish the most direct evidence of the formation of tubercles by inflammatory action. I have no doubt of being able to form a series of preparations, in which a gradual transition shall be obvious, from the true hepatized condensation of the lung, through the intermediate changes of structure to which Laennec has given the name of grey hepatization, and of tubercular infiltration, to that kind of disorganization which consists for the most part of tubercles; and I am much confirmed in my opinion as to the close analogy of these structures, by finding that the researches of Andral have led him to the belief, that the appearance to which Laennec gave the name of 'Tubercules infiltrés,' is always to be regarded as the effect of a chronic inflammation."* 286.

The following history may form a good illustration of the Professor's principle, and as none of the same kind are detailed in his paper, we will, no doubt, be excused for recording it very concisely. A gentleman, who had reached the middle period of life, became rather poorly without knowing of any adequate cause, but suspected he had received cold. The symptoms were those of a low degree of fever, quick pulse, increased temperature, thirst, general sense of uneasiness, slight dyspnœa, and a trifling cough, with loss of appetite. In a few days, small tumours, varying in size, appeared upon his skin, and although a few were visible on different parts of his body, his arms were their principal seat. They were hard and slightly painful, did not seem inclined to suppurate, and were decidedly of the tubercular form. After their eruption the dyspnœa increased, the cough became more frequent, and, in about two weeks after, death put an end to the symptoms. On opening the body, the lungs were found slightly inflamed, and a few adhesions existed between the pleuræ; but the principal morbid curiosity consisted of a very large crop of miliary tubercles, which were equally diffused throughout the parenchyma of the lungs, and occupied the greater part of it. On comparing them with the granular bodies upon the arm, the resemblance was striking, and all who

* "See Lombard's Thesis, p. 32."

were present at the inspection of the body expressed their conviction, that no essential, if any, difference existed between them, when they considered the difference of texture in which they were found. The Professor likewise gives a very interesting case at the 421st page of the first volume of these Transactions, where a child, æt. 21 months, complained of the usual symptoms of pulmonary disease, with inability to lie on *the right side.* On dissection, the *right lung* was found generally adhering to the ribs, and "on examining the exudation on the pleura, which formed the intimate adhesions, it appeared to consist almost entirely of a very great number of small solid tubercular masses; in a few places only it had, to a small extent, the usual diffused form of an inflammatory effusion, and the matter composing this diffused effusion appeared to be identical with that which had the form of tubercles." Dr. Abercrombie examined them, and found that they became hard and opaque on the application of boiling water, as do ordinary tubercles in their incipient state. In these two instances we have tubercular matter formed under circumstances which render it unquestionable that they were *secondary* structures, and, from the symptoms of inflammation which accompanied their appearance, and the other effects of inflammation which were discovered in conjunction with them after death, we presume there can be no doubt as to their origin.

But, in the preceding cases, we have various results of phlogistic action, and they may, therefore, be objected to as not unequivocal enough, since many of the symptoms may have arisen from the change of structure which existed *independently* of the tubercles. The following history is encumbered with no such difficulty.

A young man, æt. 16, rather delicate, began to complain, on the 6th of January, of cough, copious puriform expectoration, pain under the sternum, difficulty of lying on the right side, and dyspnœa; pulse 140, soft; tongue red; thirst; skin hot and moist; face flushed; lips livid, and aspect anxious. Five weeks previously he had been exposed to cold, immediately after which rigors and other febrile symptoms appeared, and since that period he never was exempt from cough and dyspnœa. Ten ounces of blood were taken from his arm, which produced faintness, but no relief; a blister was applied without improvement, and draughts, with æther, digitalis, and expectorants, were given without benefit. He became delirious the next day, his respiration continued short and quick, and he died on the evening of the 9th.

*Dissection.* Slight deposition of lymph on the pleuræ of both sides; the lungs crepitated, but when cut into, were found "studded throughout with an immense number of very small tubercles, exactly corresponding to Laennec's description of 'tubercules miliares.'" The Professor had never before seen so many in one case, nor so uniformly deposited; one only was found in a state of suppuration, but in the upper part of the lungs they were becoming white, *(tubercules crus,)* and of a large size. Besides this appearance, however, there was nothing to be seen, the parenchymatous substance being healthy, with the exception of a few patches of hepatization." P. 287—90.

Before this young man was placed under the care of Dr. Alison, it was found that he had been two months in the hospital, labouring under similar symptoms; that they had also been excited by exposure to cold; that they had been *completely removed* by a blister and purgatives in the course of a few days, and that, before that period, he had never felt any pectoral uneasiness. It must be admitted by an impartial reader, that the symptoms detailed in this case were precisely those of pneumonic inflammation, that the remote cause was the one most likely to excite it, and that had not the lungs been inspected, seeing that the stethoscope had not been applied, the patient must have been regarded as having died of the consequences of a neglected pneumonia. But dissection discovers no phlogistic products, if the tubercles be denied such an origin. There is neither inflammation, nor consolidation of the parenchyma, adhesion of the pleuræ, nor serous effusion. The only appearances are a trifling layer of lymph on the surface of the lungs, and a universal and uniform crop of tubercular deposits. What, then, occasioned such distressing, such constant dyspnœa, such lividity of the face, such velocity of the pulse, and such painful decubitus? Certainly not the cob-web effusion of lymph, nor the few isolated specks of consolidated parenchyma! Had nothing morbid existed beyond these the man might have lived to the age of Nestor. The tubercles can alone account for them, and the first question still recurs, when and how were these tubercles formed? Did they exist before the boy's exposure to cold, and were they elicited by its excitement into action, or were they formed by the attack of inflammation which this excitement evidently occasioned?

It will be hardly insisted on by any modern pathologist, who knows to what an extent such a large crop of tubercles as existed in this case will derange the functions of the circulating and respiratory organs, that they could all have existed before the attack of inflammation which occasioned death. If they did exist before it, what was the cause of death? Was it the shred of lymph, or the scrap of hepatization? The lungs were not in a worse condition when the man died than before it; and if all the tubercles were formed before his illness which were discovered after it, we have not only a severe attack of pneumonic inflammation without any palpable affection of the lungs, but we have death ushered in by every symptom which can betoken pulmonary disease, without any disease being discoverable (at least of importance) beyond what had existed for sixteen years of uninterrupted health. When we consider, then, the absence of every pulmonic symptom previous to this man's first attack of illness—the nature of the exciting cause—the suddenness of the attack—the facility with which the inflammatory symptoms were subdued in the first instance, and the character of the remedies by whose agency they were removed—their speedy return upon exposure to the same cause, after they had been entirely dispelled—their obstinacy under every form of treatment, and the rapidity of their progress to dissolution—the absence of every phlogistic product of any weight, and the number of tubercles, almost all of the same size, and of that kind which the best pathologists believe to be indicative of the first stage of their formation; we may conclude, we presume, with tolerable safety, that few or no tubercles existed in this case previous to the first attack of inflammation, that by this attack the lungs became weakened and disposed to disease, that from a fresh exposure inflammation re-commenced, and, in the course of a few weeks, impacted the lungs with so many tubercular deposits, that the respiratory and circulating functions became so injured as to destroy life.

The Doctor adduces a very similar case from Bayle, and exposes, in a very just critique, the insufficiency of the author's explanation of death on the supposition that the tubercles which were found, and which, as in the above history, were all that was found of importance, had existed long before the injury was received upon the chest, and that the fall had merely given a shock to the lungs which deranged their functions and produced death.

"The affections of the intestines and mesenteric glands, which are so apt to occur in the course of phthisis, appear to me to furnish frequently unequivocal examples of tubercular deposition resulting, I do not say from active inflammation, but at least from increased vascular action and congestion of blood, which may be properly called chronic inflammation.

"It has been indeed supposed by Laennec, that the colliquative diarrhœa of phthisis depends in general on the existence of tubercles in the intestines, and of ulcerations formed in these tubercles; and it is certain, that in those patients who are affected with this diarrhœa long before death, these tubercles and ulcers in the intestines, as well as more or less of the tubercular degeneration of the mesenteric glands, are very generally found. But I have had several opportunities of observing, in cases where the colliquative diarrhœa, although well marked, has been of shorter duration before death, that the mucous membrane of the intestines has presented no morbid appearance, except general vascularity; and in other cases there have been only a few tubercles at the most vascular points; from which I conclude that the numerous tubercles and ulcers found in those cases where the diarrhœa has lasted long, are the consequence, not the cause, of that increased vascular action of the part, which produces the diarrhœa. And on examining the mesenteric glands in persons who have died of phthisis after various degrees of the colliquative diarrhœa, or the different mesenteric glands in the same subject, it has appeared to me obvious that the first change which takes place in them, is an increase of size and of vascularity; that the first tubercular deposition occurs at minute points in glands already thus altered, and that it is only by the gradual extension of these deposits, in glands larger and more vascular than natural, that the conversion of the glandular substance into the scrofulous cheesy matter is effected. The first part of these changes in the abdomen, in phthisical persons, is I think generally attended with pain, though not constant, and not always severe." 297.

A boy, æt. 12, who had been ever free from any pectoral complaint, was severely injured *on the lower part of the left side of the chest* by a fall—so much so, that it confined him to bed for several weeks, he lost his strength, his breathing became short, and he seldom went out of doors. He died of small-pox, and on dissection no disease existed within the chest, *except under the very part which sustained the fall. The lower lobe of the left lung* adhered to the pleura costalis, and was completely condensed, partly by red hepatization and partly by tubercular formations, which were yellow, and of the consistence of cheese. 297-8.

Now, it must be admitted by those who deny the inflammatory origin of tubercles, that the circumstance of that portion of the lung which received the injury being the only one which contained these bodies, was, at the least, a very strange coincidence, and when we say that such a coincidence is *possible*, we grant as much as even the uncertainties of chance can require; for we know that the upper portions of the lungs are the most obnoxious to tubercles, and the lower most subject to condensation; so that, in this instance, the probabilities of the coincidence are very much diminished, and, although evidence is not irrisistible so long as it is obnoxious to a possibility of error, the chances are very much in favour of Dr. Alison's doctrine; and, could a sufficient number of cases of a similar character be collected, it would be an unaccountable scepticism which would resist their testimony.

Something analogous to such cases in miniature may be found in the facts, that masons, pin-makers, needle-grinders, feather-dressers, and such mechanics as inhale an atmosphere impregnated with matter which may irritate the lungs, are peculiarly exposed to tubercular phthisis. Some of Portal's pupils, who were barber-surgeons, found it necessary, from a similar cause, to leave off hair-dressing; and Wepfer relates that the workmen, who were occupied in a millstone quarry at Waldshut on the Rhine, became consumptive, if they remained in it for a year, and died unless early assistance was provided. How can the professions of such men operate in diseasing the chest, and why are they especial victims to consumption? There is no very plausible solution to these questions if we do not admit, that bodies floating in the atmosphere, and inhaled into the lungs, may excite tubercular disease. That they will excite inflammation no one can dispute, and as we do not find that, in cases arising from such causes, no tubercles are discovered upon dissection, the post-mortem results are, in this respect, similar with idiopathic cases; and, unless the absurdity be advocated, that all those who engage in such professions are not only of a scrofulous habit, but have been the victims of tubercular disease before they adopted them, the historic fact, that such workmen, and such workmen only, are peculiary liable to consumption, involves a proof, amounting almost to the certainty of demonstration, that these structures may be elicited by inflammatory action.

This point has, however, been rendered still more certain by experiment. Cruveilhier injected mercury into the femoral artery of a dog, which was killed a few days after, when crowds of miliary tubercles were found regularly formed in the injected limb, and a small globule of mercury constituting the nucleus of each. Another dog was taken, an opening was made into his trachea, and ℥ij. of mercury were injected through this aperture into his lungs. The greater part of it was coughed up, but, in the course of a month, after great emaciation and pectoral disturbance, the animal died. The lungs were found stuffed with solitary and agglomerated bodies, having all the characters of miliary tubercles, (*Nouvelle Bibliotheque Medicale, for Sept.* 1826.) Similar experiments were instituted by Dr. Kay, and they were attended by similar results. As the language in which they are detailed is concise, and the evidence which they furnish is convincing, we will not attempt a condensation.

"A small globule of mercury was introduced into the trachea of each of the rabbits which were the subjects of the experiments, by small incisions, which soon healed. This produced at first much coughing, which occasionally returned afterwards; but the animals did not appear much incommoded, and took food well; their breathing, however, appearing rather hurried.

"The first rabbit was killed eight days after the introduction of the mercury; and it is singular that the appearance of clusters of tubercles was more distinct in this than in any of the others. These lungs are preserved, but the colour of the tubercles does not now contrast with that of the pulmonary substance as before the lungs were put in spirits, and several of the clusters were destroyed in examining them minutely. The appearances of several of the clusters were so exactly those of tubercles in their early stage, that my learned colleague Dr. Monro, and several other friends, gave them that name without hesitation, before they were aware how they were produced; but on cutting into them, each contained in its centre a minute globule of mercury.

"It is to be observed farther, that, in several parts of these lungs, there was partial hepatization.

"The appearances found in the other rabbits are accurately described by Dr. Kay in the following paper:—

"'The *second* rabbit, after appearing drowsy and oppressed for a day, was found dead eight days after the operation.

"'The upper lobe of the left lung was discovered hepatized in the whole of its anterior border, through which were scattered small granular bodies, of a yellowish colour, each containing in its centre an extremely small globule of mercury. They had all the external characters of ordinary tubercles. In the centre of this lobe existed a cavity, containing a soft caseous substance, surrounded by a firm membranous cyst. It was about two and a half lines broad, and three in length. Large globules of mercury escaped from this cavity. In the posterior part of this lobe was an extremely small cyst, containing neither tubercular matter nor a mercurial globule.

" 'The lower lobe of the left lung was also hepatized in the whole of the anterior border, through which numerous small, granular, and apparently tubercular bodies were deposited, containing generally a small globule of quicksilver in their interior. The superior lobe of the right lung was, in some portions, hepatized. A greyish, and apparently tubercular, substance was infiltrated into portions of the pulmonary texture. In the anterior part of this lung, existed a large encysted cavity, filled with a soft, yellowish, granular mass, of a caseous consistence, resembling the matter of softened tubercle. The cyst was distinct, apparently well organised, being of firm texture; and the cavity was capable of containing a large pea. In the lower lobe of this lung, tubercular deposition also existed, surrounded by hepatized structure. Several bronchiæ were much dilated, and a similar substance was deposited round their parietes.

" 'The other rabbits were killed four weeks after the operation.

" '*Third Rabbit.*—In the upper lobe of the left lung, a large globule of mercury was found imbedded in a quantity of soft and greyish lymph, surrounded by a distinct cyst. Many globules of mercury were found scattered in the lungs, without either hepatization or deposition of lymph. The lung, however, appeared emphysematous, and some dilated bronchiæ were observed. In the right lung the appearances were similar. The globules generally were found in the thin border of the lung, and appeared to have excited little inflammation there. Some partial condensation, and a few granular bodies resembling tubercles, were observed.

" '*Fourth Rabbit.*—Much hepatization had been produced in this lung. Dilated bronchiæ were observed in many portions, and in the extreme thin border of the tissue many globules of mercury existed, unsurrounded by hepatized structure or lymph, but apparently contained in enlarged cells. In the centre of the lung, as before, they were, in some cases, surrounded by lymph, which had the form of tubercles.

" '*Fifth Rabbit.*—The general appearances in this lung resembled those described in the others; but, at the extreme thin border of the lung, a row of globules of mercury existed, not simply contained in air-cells, but surrounded each by a very evident thin deposition of lymph.' " 303.

We believe that tubercles have hitherto been discovered principally, if not exclusively, in mammiferous animals and birds; in the first class the herbivoræ are principally affected, and we know of no instance in which they have been found in dogs. This fact, consequently, renders these experiments very satisfactory; and if further proof were necessary, that the tubercles found in the subjects of these experiments could not have existed prior to their performance, it will be seen in the circumstance that a globule lay in the centre of each, and the same fact renders it certain that these bodies were formed by the mercurial irritation. It is likewise clear that they were not only produced by the irritation of the quicksilver, but in consequence of inflammation kindled by this excitement; because we have the ordinary proceeds of inflammation, in addition to these formations—viz. hepatization and effusion of lymph. There can be, therefore, we presume, no plea for resisting the inference that these bodies, supposed by Cruveilhier, Monro, Alison, Abercrombie, and Kay, to be tubercles, were formed during the experiments, and in consequence of inflammation produced by them.

The Dr. does not wish it to be understood, that he considers the tubercles thus formed may pass through the same stages, and assume in their progress to maturation, the same characters as purely scrofulous formations. His object is to prove that bodies, to all appearance similar, may be excited by ordinary inflammation in ordinary systems,—and, that, though they may differ in some of their externals from common tubercles, they dispose to the same disease, and lead to the same end. It is, no doubt, very difficult to show how ordinary inflammation, applied *in a peculiar way*, can give rise to a morbid product, similar to that elicited by scrofula; but, if the reader be now satisfied from the evidence advanced, that such is in reality the case, our inability to explain the phenomenon is no argument against the occurrence of the phenomenon itself. The following is the Professor's explanation, which, however, we must admit, is not quite satisfactory.

"It was stated already, as the result of the observations of Andral, that the conditions which appear most requisite, in order that inflammation may generate tubercles in the living body, are the *long duration* and *slight intensity* of that inflammation. It is highly probable that the scrofulous diathesis disposes inflammation to terminate by tubercular deposition, simply by giving to it these characters,—keeping it up long, and not permitting it to rise high.

"Now, it is very easy to conceive, that the continued irritation of a minute globule of mercury imbedding itself in a portion of pulmonary substance, or the repeated irritations of particles of sand inhaled, may furnish just the same condition to the inflammation which they excite,—as is given in other cases by the peculiar strumous diathesis,—rendering the inflammation long continued, but of slight intensity. When this cause is habitually applied for a long time, and during youth to the human species, as in masons, I believe that very few cases exist, even of constitutions previously strong and healthy, in which more or less of tubercular deposition is not excited." 305.

That a *slow* and a *slight* inflammatory action may be favourable to the developement of these bodies may be true; but, if so, how did the mercury, either in the experiments of Kay or Cruveilhier, dispose to such a species of action? In one of the instances above quoted, the dog was killed *some* days after the experiment; in another, the animal died of the effects of it in a *month;* and the second rabbit of Kay was found dead *eight days* after the operation. The inflammation which was excited in these cases was neither chronic as to time nor intensity, and the disorganizing effects which it produced were such as indicated no trifling degree of action. When a scrofulous predisposition exists, we believe that *any* excitement may call forth tubercular matter, and the only reason, which we can conceive, why a *trifling* and *tedious* action may be more conducive to such deposits than one which is *rapid* and *intense* is, that time is given for the development of the scrofulous taint, and that the stimulus is strong enough to effect this development, but not acute enough to destroy life.

"I think the facts stated in this paper, and in the latter part of my former one, may be regarded as sufficient evidence of the proposition, that *scrofulous tubercles may be, and often are, deposited in consequence of inflammatory action;* and therefore, that as, on the one hand, scrofulous disease may be in many cases prevented by applying the *tonic regimen* to persons of feeble constitution, but not yet affected with actual disease; (the Professor here allows some inaccuracy to creep into his language; for had *the persons of feeble constitution been affected, how could preventive medecine prove beneficial?*) so, on the other, they may also be frequently prevented by the early and prudent use of the *antiphlogistic remedies* in those in whom the slight inflammatory complaints so often preceding them have already appeared.

"This general conclusion does not differ from that which I believe the greater number of practitioners have been induced to form, from their own practical observation; but it seems to me of great importance to have attained to it by pathological inquiries, founded on fatal cases,—because, until the pathological question as to the frequent dependence of tubercles on inflammation is determined, no observations on the effect of the antiphlogistic remedies, where tubercular disease is apprehended, —if made on cases that terminate favourably,—can be held to be conclusive on the subject." 307.

There is a sentiment expressed in this extract which we feel some reluctance in adopting. That tubercles may be *created* or formed *de novo*, by the action of ordinary inflammation, and that such tubercles do not differ materially from the unsolicited products of a strumous habit, we believe, and have endeavoured to prove; yet, we question very much, in the present state of the subject, whether they should be called, or considered *scrofulous*, unless we admit, with Dr. Young, that, "every person may be said to possess more or less of a scrofulous taint." Our knowledge of scrofula, and our analyses of these formations are yet in a very imperfect state, and, perhaps, we should, in the elementary stage of the present inquiry, rest satisfied with the ultimate and practical fact, that bodies may be formed by inflammation which bear all the appearance of ordinary tubercles, and lead to the same result.

Whether the author's views, or ours, as to this point be correct, we leave future investigation to determine; the general question suffers nothing from our difference, and will not be altered by the decision; for if the proofs now collected be considered sufficient to establish the occasional origin of tubercles, and, of consequence, consumption, in inflammation; and, if it be true that pure inflammation, *properly moderated as to duration and degree*, can elicit such bodies in untainted systems, the practical conclusion to be drawn will be most important. The *strumous race* can no longer be regarded as the only victims of tubercular phthisis, and we are henceforward to employ, with discriminating caution, that tonic system of therapeutics on which we have hitherto generally depended. Whether we believe that the rudiments of these tubercles exist in an invisible and innocuous state prior to the inflammation, or maintain that they are its sole product—whether we imagine that a scrofulous predisposition is necessary to favour their deposition, or that a certain quantum of inflammatory action can produce them under any circumstances—the deduction remains unchanged in kind, and unabated in degree. The great object is to know that pulmonary inflammation, however excited, may develop tubercular matter, and thus sow the seeds of one of the most fatal and deceitful diseases with which we have to struggle; and, if this point be satisfactorily established, it matters little whether this inflammation must be aided by a predisposition, whether it can work ex primis, or unfold into importance seminal principles already in existence. Dr. Alison's two valuable papers upon this important question we consider highly interesting; and, although the evidence with which he has supported his principles, may not amount to demonstration, it will be difficult to overturn the arguments he has advanced, or explain away the facts he has recorded. More light—much more light may, however, be cast upon this interesting subject, and we look forward, with sanguine anticipation, for the appearance of more "additional observations" from the same pen, when we shall feel happy in resuming our critical labours, and adding our mite to the treasury of science.

